# Supplementary material for: Resolving the full spectrum of human genome variation using Linked-Reads
Source: Genome Res. 2019 Apr;29(4):635–45. doi: 10.1101/gr.234443.118 (PMC6442396; doi:10.1101/gr.234443.118)
Supplement: Supplemental Material [file supp_gr.234443.118_Supplemental_longranger-2.2.zip › longranger-2.2.tar.bz2!/lr2.2-public/lib/go/src/code.google.com/vitess/docs/user-guide/twopc.html]

Vitess / 2PC Guide


Toggle navigation

- Overview
- Guides
- Reference
- Blog
- GitHub

- #### Overview

  - What is Vitess
  - Scaling MySQL with Vitess
  - Key Concepts
- #### Getting Started

  - Run Vitess on Kubernetes
    - Custom Docker Build
  - Run Vitess Locally
- #### User Guide

  - Introduction- Client Libraries- Backing Up Data- Reparenting
        - Schema Management
        - VSchema Guide
        - Schema Swap (Tutorial)
        - Sharding
          - Horizontal Sharding (Tutorial, automated)
          - Horizontal Sharding (Tutorial, manual)
          - Sharding in Kubernetes (Tutorial, automated)
          - Sharding in Kubernetes (Tutorial, manual)
        - Vitess Sequences
        - MySQL Server Protocol
        - Vitess and Replication
        - Update Stream
        - Row Based Replication
        - Topology Service
        - Transport Security Model
        - Launching
          - Scalability Philosophy
          - Production Planning
          - Server Configuration
          - 2PC Guide
          - Troubleshooting
        - Upgrading
- #### Reference Guides

  - Vitess API- vtctl Commands
- #### Other Resources

  - Presentations- Blog- Roadmap
- #### Contributing

  - Contributing to Vitess- GitHub Workflow- Code Reviews
- GitHub

# 2PC Guide

- #### Overview

  - What is Vitess
  - Scaling MySQL with Vitess
  - Key Concepts
- #### Getting Started

  - Run Vitess on Kubernetes
    - Custom Docker Build
  - Run Vitess Locally
- #### User Guide

  - Introduction- Client Libraries- Backing Up Data- Reparenting
        - Schema Management
        - VSchema Guide
        - Schema Swap (Tutorial)
        - Sharding
          - Horizontal Sharding (Tutorial, automated)
          - Horizontal Sharding (Tutorial, manual)
          - Sharding in Kubernetes (Tutorial, automated)
          - Sharding in Kubernetes (Tutorial, manual)
        - Vitess Sequences
        - MySQL Server Protocol
        - Vitess and Replication
        - Update Stream
        - Row Based Replication
        - Topology Service
        - Transport Security Model
        - Launching
          - Scalability Philosophy
          - Production Planning
          - Server Configuration
          - 2PC Guide
          - Troubleshooting
        - Upgrading
- #### Reference Guides

  - Vitess API- vtctl Commands
- #### Other Resources

  - Presentations- Blog- Roadmap
- #### Contributing

  - Contributing to Vitess- GitHub Workflow- Code Reviews

# 2PC User guide

# Overview

Vitess 2PC allows you to perform atomic distributed commits. The feature is implemented using traditional MySQL transactions, and hence inherits the same guarantees. With this addition, Vitess can be configured to support the following three levels of atomicty:

1. **Single database**: At this level, only single database transactions are allowed. Any transaction that tries to go beyond a single database will be failed.
2. **Multi database**: A transaction can span multiple databases, but the commit will be best effort. Partial commits are possible.
3. **2PC**: This is the same as Multi-database, but the commit will be atomic.

2PC commits are more expensive than multi-database because the system has to save away the statements before starting the commit process, and also clean them up after a successful commit. This is the reason why it's a separate option instead of being always on.

## Isolation

2PC transactions only guarantee atomicity: Either the whole transaction commits, or it's rolled back entirely. It does not guarantee ACID Isolation. This means that a third party that performs cross-database reads can observe partial commits while a 2PC transaction is in progress.

Guaranteeing ACID isolation is very contentious and has high costs. Providing it by default would have made vitess impractical for the most common use cases.

However, it is possible for the application to judiciously request ACID isolation where critical: If `SELECT`s are performed with `LOCK IN SHARE MODE`, then you're guaranteed that the data will not be modified by anyone else until the transaction is complete.

# Configuring VTGate

The atomicity policy is controlled by the `transaction_mode` flag. The default value is `multi`, and will set it in multi-database mode. This is the same as the previous legacy behavior.

To enforce single-database transactions, the VTGates can be started by specifying `transaction_mode=single`.

To enable 2PC, the VTGates need to be started with `transaction_mode=twopc`. The VTTablets will require a few more flags, which will be explained below.

The VTGate `transaction_mode` flag decides what to allow. The application can independently request a specific atomicity for each transaction. The request will be honored by VTGate only if it does not exceed what is allowed by the `transaction_mode`. For example, `transacion_mode=single` will only allow single-db transactions. On the other hand, `transaction_mode=twopc` will allow all three levels of atomicity.

# Driver APIs

The way to request atomicity from the application is driver-specific.

## Go driver

For the Go driver, you request the atomicity by adding it to the context using the `WithAtomicity` function. For more details, please refer to the respective GoDocs.

## Python driver

For Python, the `begin` function of the cursor has an optional `single_db` flag. If the flag is `True`, then the request is for a single-db transaction. If `False` (or unspecified), then the following `commit` call's `twopc` flag decides if the commit is 2PC or Best Effort (`multi`).

## Java & PHP (TODO)

## Adding support in a new driver

The VTGate RPC API extends the `Begin` and `Commit` functions to specify atomicity. The API mimics the Python driver: The `BeginRequest` message provides a `single_db` flag and the `CommitRequest` message provides an `atomic` flag which is synonymous to `twopc`.

# Configuring VTTablet

The following flags need to be set to enable 2PC support in VTTablet:

- **twopc\_enable**: This flag needs to be turned on.
- **twopc\_coordinator\_address**: This should specify the address (or VIP) of the VTGate that VTTablet will use to resolve abandoned transactions.
- **twopc\_abandon\_age**: This is the time in seconds that specifies how long to wait before asking a VTGate to resolve an abandoned transaction.

With the above flags specified, every master VTTablet also turns into a watchdog. If any 2PC transaction is left lingering for longer than `twopc_abandon_age` seconds, then VTTablet invokes VTGate and requests it to resolve it. Typically, the `abandon_age` needs to be substantially longer than the time it takes for a typical 2PC commit to complete (10s of seconds).

# Configuring MySQL

The usual default values of MySQL are sufficient. However, it's important to verify that `wait_timeout` (28800) has not been changed. If this value was changed to be too short, then MySQL could prematurely kill a prepared transaction causing data loss.

# Monitoring

A few additional variables have been added to `/debug/vars`. Failures described below should be rare. But these variables are present so you can build an alert mechanism if anything were to go wrong.

## Critical failures

The following errors are not expected to happen. If they do, it means that 2PC transactions have failed to commit atomically:

- **InternalErrors.TwopcCommit**: This is a counter that shows the number of times a prepared transaction failed to fulfil a commit request.
- **InternalErrors.TwopcResurrection**: This counter is incremented if a new master failed to resurrect a previously prepared (and unresolved) transaction.

## Alertable failures

The following failures are not urgent, but require someone to investigate:

- **InternalErrors.WatchdogFail**: This counter is incremented if there are failures in the watchdog thread of VTTablet. This means that the watch dog is not able to alert VTGate of abandoned transactions.
- **Unresolved.Prepares**: This is a gauge that is set based on the number of lingering Prepared transactions that have been alive for longer than 5x the abandon age. This usually means that a distributed transaction has repeatedly failed to resolve. A more serious condition is when the metadata for a distributed transaction has been lost and this Prepare is now permanently orphaned.

# Repairs

If any of the alerts fire, it's time to investigate. Once you identify the `dtid` or the VTTablet that originated the alert, you can navigate to the `/twopcz` URL. This will display three lists:

1. **Failed Transactions**: A transaction reaches this state if it failed to commit. The only action allowed for such transactions is that you can discard it. However, you can record the DMLs that were involved and have someone come up with a plan to repair the partial commit.
2. **Prepared Transactions**: Prepared transactions can be rolled back or committed. Prepared transactions must be remedied only if their root Distributed Transaction has been lost or resolved.
3. **Distributed Transactions**: Distributed transactions can only be Concluded (marked as resolved).

Contact: vitess@googlegroups.com  **·**  
Announcements  **·**  
© 2017 Vitess powered by Google Inc
